# Effect of silicon application with mycorrhizal inoculation on *Brassica juncea* cultivated under water stress

**DOI:** 10.1371/journal.pone.0261569

**Published:** 2022-04-07

**Authors:** Ashutosh Srivastava, Vijay Kumar Sharma, Prashant Kaushik, Mohamed A. El-Sheikh, Shaista Qadir, Sheikh Mansoor

**Affiliations:** 1 Department of Botany, Rani Lakshmi Bai Central Agricultural University, Jhansi, Uttar Pradesh; 2 Department Genetics and Plant Breeding, Banda University of Agriculture and Technology, Banda, Uttar Pradesh, India; 3 Instituto de Conservación y Mejora de la Agrodiversidad Valenciana, Universitat Politècnica de València, Valencia, Spain; 4 Botany and Microbiology Department College of Science, King Saud University, Riyadh, Saudi Arabia; 5 Department of Botany, Womens College, Srinagar, Jammu and Kashmir, India; 6 Division of Biochemistry FBSc, SKUAST Jammu J&K, Jammu and Kashmir, India; Ghazi University, PAKISTAN

## Abstract

*Brassica juncea* L. is a significant member of the Brassicaceae family, also known as Indian mustard. Water is a limiting factor in the successful production of this crop. Here, we tested the effect of water shortage in *B*. *juncea* plants supplemented with or without the application of silicon and arbuscular mycorrhizal fungi in total 8 different treatments compared under open filed conditions using a randomised complete block design (RCBD). The treatments under control conditions were control (C, T1); C+Silicon (Si, T2); C+My (Mycorrhiza; T3); and C+Si+My (T4). In contrast, treatments under stress conditions were S (Stress; T5); S+Si (T6); S+My (T7) and S+Si+My (T8), respectively. In total, we evaluated 16 traits, including plant response to stress by evaluating peroxidase (POD), superoxide dismutase (SOD), and catalase (CAT) activity. The fresh weight (g) increased only 7.47 percent with mycorrhiza (C+My) and 22.39 percent with silicon (C+Si) but increased 291.08 percent with both mycorrhiza and silicon (C+Si+My). Using mycorrhiza (S+My) or silicon (S+Si) alone produced a significant increase of 53.16 percent and 55.84 percent in fresh weight, respectively, while using both mycorrhiza and silicon (S+Si+My) together produced a dramatic increase of 380.71 percent under stress conditions. Superoxidase dismutase concentration (Ug^−1^ FW) was found to be increased by 29.48 percent, 6.71 percent, and 22.63 percent after applying C+My, C+Si and C+Si+My, but treatment under stress revealed some contrasting trends, with an increase of 11.21 percent and 19.77 percent for S+My, S+Si+My, but a decrease of 13.15 percent for S+Si. Finally, in the presence of stress, carotenoid content (mg/g FW) increased by 58.06 percent, 54.83 percent, 183.87 percent with C+My, and 23.81 percent with S+My and S+Si+My, but decreased by 22.22 percent with S+Si. Silicon application proved to be more effective than AMF treatment with *Rhizophagus irregularis*, and the best results were obtained with the combination of Si and AMF. This work will help to suggest the measures to overcome the water stress in *B*. *juncea*.

## Introduction

*Brassica juncea (Czern) L*. is an important member of the family *Brassicaceae*; it is also known as an ’Indian mustard (AABB, 2n = 36) [1, 2]. This is a common species of amphidiploids from a cross between *B*. *rapa* (AA, 2n = 20) and *B*. *nigra* (BB, 2n = 16) [3]. It is extensively cultivated in India, Canada, Australia, China and Russia [4, 5]. The economic importance attempts were made to raise their economic and agro-important characteristics such as oil quality, oil purity, seed scale, shattered pods and pathogen resistance. However, only a few papers covered the effects of water stress on important stages of *B*. *juncea* and how to eliminate it. However, *B*. *juncea’s* water footprint is very small compared to most other Indian crops [6, 7]. Still, the emergence and sustainability of seedlings were severely hampered by severe water stress [8]. High temperatures and water tension during pod development are often considered to mitigate seed setting [9].

A major environmental factor limiting plant growth and crop quality is soil water availability [10, 11]. Water deficiency is due to surface water depletion (drought) or its absorption issue (physiological drought) [12]. Water is in the soil solution in this case. Still, plants cannot use it since there are certain physiological considerations such as elevated levels of salt (salinity), surplus water (floods) and low temperatures [13, 14]. Therefore, all of these factors influence water stress and changes in cell waters. Water potential decreases, and plant cell turgor decreases [15]. This interrupts most vital processes and reduces rates of production. Water deficit affects photosynthesis, absorption and transmission of essential nutrients and causes overproduction of ROS–species of reactive oxygen (O2-, 1O2, OH, H2O2) [16, 17]. These very reactive molecules cause extreme metabolic disorders and degradation of the membrane [18]. Silicon has been developed to reinforce cell walls and provide mechanical support to monocots and pteridophytes (under-understanding of dicots) by enhancing suberisation [19, 20].

Silicon (Si) application enhances the growth and increases plant tolerance to various abiotic stresses [19, 21, 22]. Soil content of Si can vary considerably between 1.0 and 45 percent dry weight [23]. While all plants have Si, Si levels differ substantially among species, ranging from 0.1 to 10% of the dry weight above ground [24, 25]. Monocots plants usually consume Si actively, whereas most dicots plants absorb Si passively [26, 27]. While Si is not an effective ingredient for higher plants, exogenous Si application has positively affected plant growth under abiotic stress [27, 28]. However, the advantages of Si are negligible or sometimes non-existent except for stresses of some sort. Silicon treatment has been studied under water stress in some Si accumulators, like rice, maize, wheat and sorghum [29–31]. When Si is added, photosynthesis and associated carboxylase activities are increased under field drought conditions, as observed in wheat [32, 33]. In maize, Si addition can increase K and Ca levels, which indicates Si’s essential role in plant mineral balance [34].

*Glomus spp*. spores such as *G*. *mosseae* and *G*. *intraradices* are the main root colonizers in saline soils [35, 36]. AM colonization enhances plant resistance to salinity, improves plant productivity, increases nutrient absorption [37], maintains ion equilibrium and facilitates water uptake [38]. Therefore, it would be important to study AM symbiosis’s action in increasing the consumption of Si host plants and their cumulative position in salt tolerance. The hemicellulose of cell-walled connected with Si improved structural flexibility, evidently advantageous in water deficits [39, 40]. Furthermore, plant biological silicification, involving apoplastic polymerization of silicic acid, helps to form a silica barrier [41], which may reduce biotic and abiotic stress, prevent pathogenic contamination and penetration into plants potential toxicants such as aluminium (Al), manganese (Mn), cadmium (Cd), zinc (Zn) and sodium (Na) [42–44]. In root endodermis and exodermis, Silicon has helped in forming the Casparian band [20, 45]. Lignin and suberin based genes transcription were also activated [46]. These components can shape Na+ transport barriers in roots correlated with increased rice salt tolerance [47].

In this direction, Si’s effects on transpiration vary with organisms and environmental factors. While Si inoculation increased transpiration in rice under both drought and salt-stressed conditions, it decreased the unstressed transpiration of rice [48]. The findings were similar in drought-stressed wheat and sorghum, while Si decreased transpiration in dry maize, and no effects were recorded on cucumber [20].

Such heterogeneity indicates divergent techniques among different plants, matching water absorption rates and water loss at the leaf stage. These methods and Si responses need further study and discovery. However, pathways that influence aquaporin’s speech and function through the Si diet have yet to be resolved [49]. In addition to affecting hydraulic conductance and water transit by modulating aquaporin expression/activity, Si may impact water transport by modifying cell osmotic potential with enhanced osmolyte accumulation (e.g., proline, soluble sugars, inorganic ions, etc.) [50]. Therefore, the present study was carried out to study the role of Si application and mycorrhizal fungi for *B*. *juncea* plants cultivated with and without water stress.

## Material and methods

### Experimental layout

Field experimentation was carried out at Agriculture Research Farm, located at a latitude of 29°95ˈ North and longitude of 76°82ˈ from October to March of 2016–2017 at the temperature of 30±4°C (day) and 20±5°C (night). The plot was ploughed to make uniform topography using sandy-clayey loam soil employing a randomized complete block design with three replicates. Two irrigation regimes, including control (irrigation two times, one at 50% flowering stage and another one at 50% siliquae formation time), and total stress (no irrigation), were followed. B. *juncea* cultivar (RH-749) fertilized with recommended fertilizer dose of NPK for the treatment with silicon. Water stress was developed by withholding irrigation during the vital development stage (siliquae and flower initiation phases). The seeds were obtained from the oilseed section of CCS Hisar Agricultural University, Haryana. Physio-biochemical analysis of the experimental soil showed that it contained; sand 80.32%, silt 6.11%, clay 13.18%, organic matter 0.79%, total nitrogen (N) 110.15kg/ha, phosphorous (P) 7.59 kg/ha, K 439.61 kg/ha, and S 106.49 kg/ha. The pH of the soil was slightly basic at 7.9.

### Silicon and arbuscular mycorrhizal fungi treatment

The salicylic acid solution was sprayed at a concentration of 150 ppm at the emergence, flowering, and siliqua stage of the *B*. *juncea* plants. Whereas *Rhizophagus irregularis* at a CFU count of 100 spores/g was procured from (M/S Shri Ram Solvent Extractions Pvt. Ltd., India). After mass multiplication, 100 g per plant is mixed with the field soil before transplanting.

There were eight treatments, i.e., 4 under regular irrigation and 4 under water stress. The treatments under control conditions were control (C, T1); C+Silicon (Si, T2); C+My (Mycorrhiza; T3); and C+Si+My (T4). Whereas treatments under stress conditions were, S (Stress; T5); S+Si (T6); S+My (T7) and S+Si+My (T8), respectively.

### Plant characterization

The root to shoot length was measured on a meter scale, whereas whole plant height was estimated in cm at the blooming stage (day 63). The roots were then separated from the shoot, blotted, and subsequently weighed to record their fresh weight (FW) and then placed in an oven at 80°C overnight and weighed again to record the respective dry weight (DW). The leaf area was determined by utilizing a portable leaf area meter (Systronics 211, Ahmedabad, India), as per the manufacturer’s instructions. Plants were harvested and taken for sampling from each plot. Leaves were removed from the stem, and fresh weight was noted (FW). Then they were oven-dried at 70°C overnight for weighing dry weight (DW). This is done to evaluate Leaf Water Content using [(FW–DW)/FW] ×100.

The amount of chlorophyll present was determined by taking two wavelengths, i.e., 620 and 940 nm, with a CL-01 Chlorophyll Content Meter (Hansateh, Norfolk, UK). Whereas the method used to determine the membrane stability index (MSI) is defined elsewhere [19]. Using the sodium chloride reference standard, the osmotic potential was determined from the 3rd fully expanded leaf. Electrolyte leakage was measured by Dionisio-Sese and Tobita [51]. Furthermore, we evaluated the plant response to stress by evaluating peroxidase (POD), superoxide dismutase (SOD), and catalase (CAT) activity based on our previously defined protocol [52].

### Data analysis

The data was collected from five plants that were comparable in appearance from each treatment, with the exception of the border plants. Each treatment’s mean values were submitted to an analysis of variance (ANOVA) in order to identify differences among the treatments. Futher, the statistical significance of differences between treatment means was determined using Duncan’s multiple range test (DMRT) for comparison of variance separated with (least significant difference) (LSD) as a post hoc test. All the statistical analysis were performed using JASP computer program (version 0.14.1.0). Whereas, the estimation of Pearson´s correlations coefficients was performed using the R platform (R Core Team 2015).

## Results

### Variation for characters

Variations in different traits of *Brassica juncea* were investigated after treatment with silicon and Mycorrhizal Inoculation under water stress and recorded in Table 1. The fresh weight was found to increase by only 7.47% and 22.39%, respectively, when treated with mycorrhiza (C+My) and silicon (C+Si) but increased drastically by 291.08% after treatment with both silicon and mycorrhiza (C+Si+My) (Table 1). The treatments were found to be more effective under conditions of stress which produced a significant increase of 53.16% and 55.84% in fresh weight when treated separately with mycorrhiza (S+My) and silicon (S+Si), respectively, and a dramatic increase of 380.71% when treated with both silicon and mycorrhiza (S+Si+My) (Table 1). Similarly, the dry matter content increased by 37.5%, 30.78%, and 194.67% with C+My, C+Si, and C+Si+My, respectively, and by 52.7%, 100.67%, and 261.48% with S+My, S+Si, and S+Si+My respectively (Table 1). Further, the root length exhibited a maximum increase of 30.23% with C+Si+My, followed by 15.77% with C+My, and 2.99% with C+Si. However, under conditions of stress, the treatment with silicon (S+Si) produced a maximum increase of 33.05% in the root length, followed by treatment with S+Si+My (28.58%) and S+My (14.8%) (Table 1).

**Table 1:** Variation among the different treatments for morphological traits of *Brassica juncea* when treated with silicon and mycorrhizal inoculation of under water stress in the influence of silicon and mycorrhizal inoculation.

Treatments	Fresh weight (g)	Dry matter (g)	Root length (cm)	Leaf area (cm^2^)	Plant height (cm)
C	22.33±1.53c	4.32±0.07d	16.67±0.65c	93.33±1.53cd	96.67±1.50c
C+My	24.00±1.00c	5,94±0.30c	19.30±1.00b	117.21±3.30b	104.67±1.48b
C+Si	27.33±0.85c	5.65±0.38c	17.17±0.96c	112.34±5.71b	105.67±1.71b
C+Si+My	87.33±4.51a	12.73±0.57a	21.71±1.02a	125.20±2.04a	112.03±1.95a
S	15.67±2.08d	2.96±0.08e	14.52±1.20d	71.33±0.58e	65.67±2.52f
S+My	24.00±2.00c	4.52±0.41d	16.67±1.35c	88.12±3.54d	73.67±2.52e
S+Si	24.42±1.06c	5.94±0.38c	19.32±1.45b	117.00±3.04b	104.67±1.76b
S+Si+My	75.33±5.77b	10.70±0.52b	18.67±0.65b	99.33±6.51c	89.67±1.83d

*Means within the groups are significantly different based on Duncan´s mean range test.

In the case of leaf area, it was observed that silicon and mycorrhizal inoculation increased by 25.58%, 20.36%, and 34.14% with C+My, C+Si, and C+Si+My, respectively, while it recorded an increase of 23.54%, 64.02%, and 39.25% with S+My, S+Si, and S+Si+My respectively (Table 1). With regard to plant height, the increments recorded under normal conditions were 8.27%, 9.31%, and 15.89% with C+My, C+Si, and C+Si+My, respectively. In contrast, the corresponding values observed under water stress conditions were 12.18%, 59.38%, and 36.54% with S+My, S+Si, and S+Si+My, respectively (Table 1).

However, the percentage of relative water content did not change significantly; it decreased by 2.72% when treated with C+My but increased by 2.51% and 3.67% with C+Si and C+Si+My, respectively (Table 2). Further, it increased by 2.1%, 8.92%, and 8.43% after treatment with S+My, S+Si, and S+Si+My, respectively. Likewise, the percentage of membrane stability index increased nominally by 0.02%, 3.06%, and 6.27% under the influence of C+My, C+Si, and C+Si+My respectively, and by 2.6%, 10.37%, and 7.14% with S+My, S+Si, and S+Si+My respectively (Table 2). Although the electrolytic content increased by 1.51% after treatment with C+My, it decreased by 1.7% and 3.65% when treated with C+Si and C+Si+My, respectively (Table 2). Further, the maximum reduction in electrolytic content was observed with S+Si (13.45%), followed by S+Si+My (3.97%) and S+My (2.29%). In addition, the concentration of Superoxidase dismutase was found to be enhanced by 29.48%, 6.71%, and 22.63% after the application of C+My, C+Si, and C+Si+My, respectively, but treatment under stressed conditions revealed some contrasting trends, increase of 11.21% and 19.77% with S+My and S+Si+My respectively, and decrease of 13.15% with S+Si (Table 2).

**Table 2:** Variation among the different treatments for different stress tolerance indicator traits of *Brassica juncea* when treated with silicon along with mycorrhizal inoculation of under water stress in the influence of silicon and mycorrhizal inoculation.

Treatments	Relative water content (%)	Membrane stability index (%)	Electrolytic content (%)	Superoxidase dismutase (Ug^−1^ FW)	Peroxidase (Ug^−1^ FW)	Ascorbate (Ug^−1^ FW)	Catalase (Ug^−1^ FW)
C	81.38±0.15b	66.89±1.28c	24.58±1.06e	14.89±2.27e	9.31±5.80d	5.14±2.13c	3.88±1.66e
C+My	78.66±1.50c	66.91±1.20c	26.09±0.57d	19.28±3.07d	16.30±3.60bc	9.43±1.70bc	7.08±1.63cd
C+Si	83.89±1.27a	69.95±0.77b	22.88±0.65f	15.89±2.64e	10.66±5.86d	5.96±1.57c	4.57±1.30de
C+Si+My	85.05±1.34a	73.16±1.04a	20.93±0.75g	18.26±3.43d	12.14±3.91cd	7.27±2.31c	5.66±1.65de
S	69.74±0.46e	56,54±1.76f	39.54±0.81a	22.2±4.04c	17.11±5.84b	9.83±1.22bc	9.15±1.13bc
S+My	71.84±0.52d	59.14±1.50e	37.25±0.87b	24.69±3.51b	22.06±3.28a	14.33±4.32ab	11.86±0.70b
S+Si	78.66±1.54c	66.91±1.21c	26.09±0.57d	19.28±3.07d	16.31±3.60bc	9.43±1.75bc	7.08±1.72cd
S+Si+My	78.17±0.48c	63.68±1.45d	35.57±0.70c	26.59±2.91a	23.88±3.16a	15.83±5.02a	15.35±2.18a

*Means within the groups are significantly different based on Duncan´s mean range test.

Similarly, the concentration of peroxidase increased by 75.08%, 14.5%, and 30.39% with C+My, C+Si, and C+Si+My respectively, however, under stressed conditions, it increased by 28.93% and 39.56% with S+My and S+Si+My but decreased by 4.67% with S+Si (Table 2). Further, the ascorbate content, due to the introduction of silicon and mycorrhiza, increased by 83.46%, 15.95%, and 41.44% with C+My, C+Si, and C+Si+My respectively, while it increased by 45.77% with S+My and 61.03% with S+Si+My, but decreased nominally by 4.07% with S+Si (Table 2). The catalase content recorded increments of 82.47%, 17.78%, and 45.87% when treated with C+My, C+Si, and C+Si+My respectively, however, when observed under water stress, it exhibited an increase of 29.61% after treatment with S+My and 67.76% with S+My+Si, but a decrease of 22.62% with S+Si (Table 2).

Finally, the total chlorophyll content (chlorophyll a and chlorophyll b) produced no remarkable variations. However, the carotenoid content indicated a considerable increase when introduced with both silicon and mycorrhiza (Table 3). The total chlorophyll content was reduced by 9.67% when treated with C+My, while enhanced by 3.81% and 8.90% with C+Si and C+Si+My respectively, however, when investigated under stress, it was found to increase by 12.82%, 30.03%, and 16.48% with S+My, S+Si, and S+Si+My respectively (Table 3). The carotenoid content increased by 58.06%, 54.83%, and 183.87% with C+My, C+Si, and C+Si+My respectively, while under conditions of stress, it increased by 23.81%, and 147.62% with S+My, and S+Si+My respectively, but decreased by 22.22% with S+Si (Table 3).

**Table 3:** Variation among the different treatments for leaf pigments of *Brassica juncea* when treated with silicon along with mycorrhizal inoculation of under water stress in the influence of silicon and mycorrhizal inoculation.

Treatments	Chlorophyll a (mg/g FW)	Chlorophyll b (mg/g FW)	Total chlorophyll (mg/g FW)	Carotenoids (mg/g FW)
C	2.91±0.14b	1.03±0.09a	3.93±0.23b	0.31±0.08e
C+My	2.67±0.06c	0.88±0.02b	3.55±0.07c	0.49±0.02de
C+Si	3.02±0.20ab	1.05±0.10a	4.08±0.30ab	0.48±0.03de
C+Si+My	3.17±0.15a	1.11±0.13a	4.28±0.30a	0.88±0.02b
S	2.08±0.18e	0.65±0.10c	2.73±0.06e	0.63±0.05cd
S+My	2.29±0.03d	0.79±0.03b	3.08±0.07d	0.78±0.03bc
S+Si	2.67±0.06c	0.88±0.02b	3.55±0.06c	0.49±0.12de
S+Si+My	2.38±0.07d	0.80±0.03b	3.18±0.12c	1.56±0.04a

*Means within the groups are significantly different based on Duncan´s mean range test.

### Correlations

**Fig 1:**
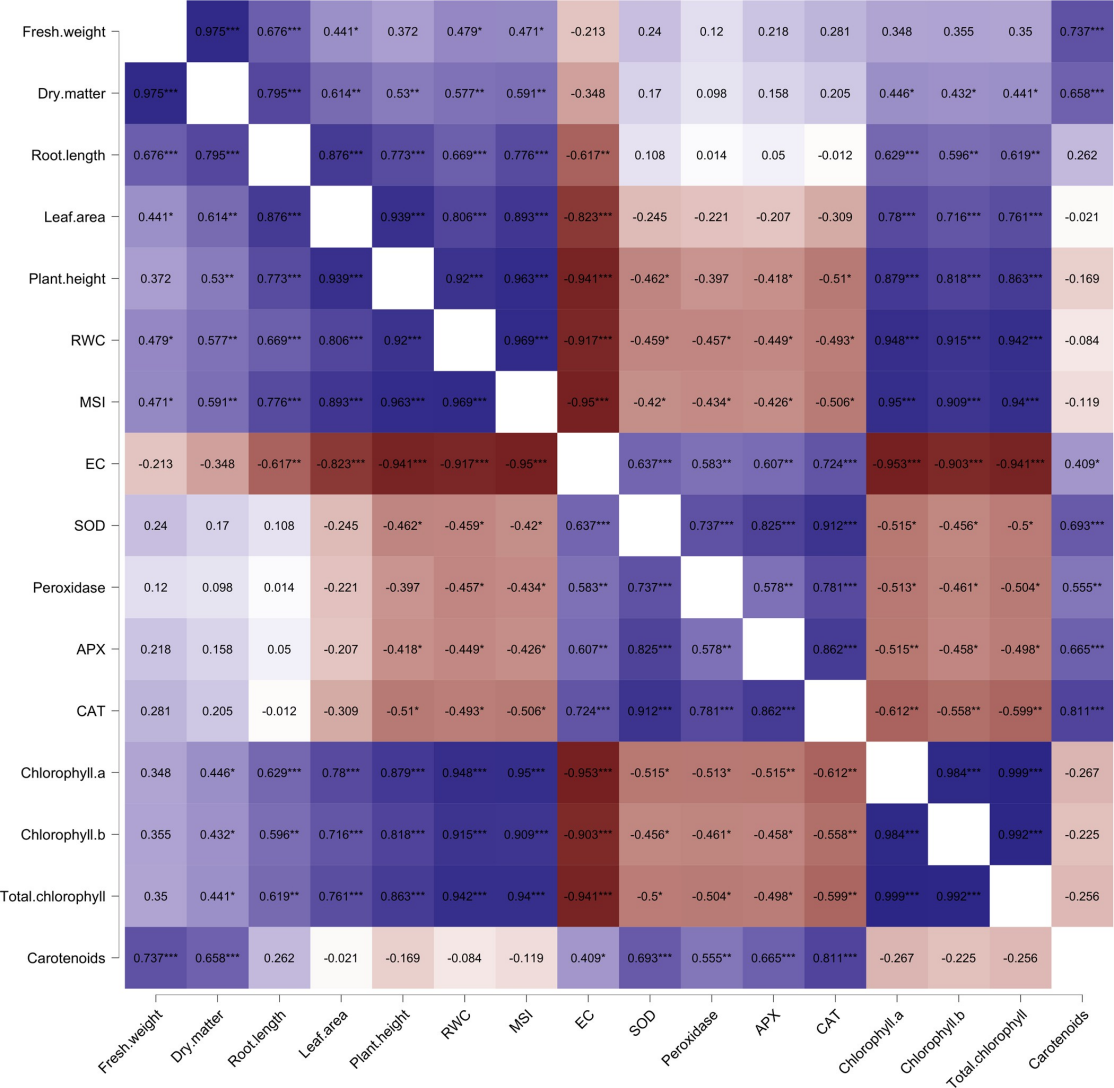
Correlations among the sixteen traits studied for *B*. *juncea* plants cultivated under water stress in the influence of silicon and mycorrhizal inoculation.

There were 136 correlations in total, but only 77 were found to be significant. Out of these significant correlations, 11 correlations were absolute (>0.9) (Fig 1). Dry matter was positively correlated with fresh weight (0.977), root length (0.792), leaf area (0.62), plant height (0.569), relative water content (0.682), membrane stability index (0.69), catalase concentration (0.49), chlorophyll a (0.513), chlorophyll b (0.469), total chlorophyll content (0.501), carotenoid content (0.832) (Fig 1). Further, the root length showed positive correlation with fresh weight (0.682), leaf area (0.877), plant height (0.8), relative water content (0.684), membrane stability index (0.841), superoxidase dismutase (0.453), chlorophyll a (0.655), chlorophyll b (0.577), total chlorophyll content (0.632), carotenoid (0.473).

The plant height was found to be positively correlated with relative water content (0.844), membrane stability index (0.931), chlorophyll a (0.759), chlorophyll b (0.625), and total chlorophyll content (0.721) (Fig 1). Further, osmotic potential exhibited a significant correlation with different traits, although in negative direction, dry matter (-0.452), root length (-0.707), leaf area (-0.864), plant height (-0.945), relative water content (-0.843), membrane stability index (-0.933), chlorophyll a (-0.883), chlorophyll b (-0.777), and total chlorophyll content (-0.856), except for electrolytic content (0.981) where it showed positive correlation (Fig 1). Besides, the concentration of superoxidase dismutase marked direct correlation with fresh weight (0.418), peroxidase (0.56), ascorbate (0.697), catalase (0.836), and carotenoid (0.599) (Fig 1).

However, the peroxidase concentration showed a significant correlation only with catalase (0.619), in addition to superoxidase dismutase. In case of ascorbate concentration, it was observed that there were considerable positive correlations with catalase (0.752) and carotenoid (0.557). At the same time, catalase concentration was also correlated with fresh weight (0.519) and carotenoid content (0.796), besides other traits mentioned above (Fig 1). Finally, the fresh weight, in addition to the traits discussed above, marked correlations with total chlorophyll content (0.44) and carotenoid content (0.877) also, both in a positive direction (Fig 1).

## Discussion

In arbuscular mycorrhizal fungi colonized plants, AMF symbiosis increased biomass and other morphological traits of *B*. *juncea* [53]. Arbuscular mycorrhizal fungi contributed to the overall better performance of the plants. The biomass production and pigment concentrations decreased under the stress conditions. Silicon and arbuscular mycorrhizal fungi treatment have elevated the symptoms of water stress and improved the quality of leaf water and photosynthesis, which have led to increased biomass growth. Silicon and arbuscular mycorrhizal fungi have been applied to increase the net photosynthesis rate by increasing stomach behavior. This growth can be attributed to better net CO2 assimilation and appropriate distribution of photosynthates that can stimulate root production in such circumstances [54, 55]. In the *B*. *juncea* plants, the combination of organic osmolytes, which contribute to osmotic gradients in the atmosphere, has been observed as a typical response to water stress.

However, osmo-adjustment did not mediate AMF and Si’s alleviatory effect and organic osmolyte levels decreased in +AMF and +Si plants [56, 57]. These results show that the Si-mediated increase in leaf water use was not due to a rise in water-stressed strawberry factories’ osmotic motive power. There was an increase in leaf’s relative water content (RWC) by increased water absorption capacity, which, in essence, prevented stomach closure and retained an excellent photosynthetic ability to support growth and the supply of dry matter [58, 59]. Rising volumes of antioxidant enzymes are usually expected to mitigate the stress. Arbuscular mycorrhizal fungi and silicon’s stress reduction solution may also be less expensive to increase water absorption than osmo-modification strategies. This discovery compares with our previous observation on tobacco plants that has revealed a Si-medium shift in plant water status by adding organic osmolytes, including soluble sugars, free amino acids and proline [60, 61].

Comparing arbuscular mycorrhizal fungi and silicon treatments to strawberry leaves revealed a specific strategy for changing the roots’ arbuscular mycorrhizal fungi and silicon water economies [62]. In tomatoes, the root osmotic potential was not altered in Si-treated plants [63]. In cucumbers, the role of osmotic motive force in Si-mediated water uptake was genotype-dependent [64]. These results collectively suggested optimizing water quality and capacity for osmotic stress-dependent Si-treated plants based on plant species, organisms and genotypes.

In plant-mycorrhizal interactions, nutrients are retained, and plant development is enhanced. Arbuscular mycorrhizal fungi hyphal networks and glomalin secretion help soil absorb water and nutrients [65–67]. Moreover, arbuscular mycorrhizal fungi can develop drought-adaptive strategies utilizing radical extra-hyphae and affect plant processes such as photosynthesis, root conductivity and root architecture [68, 69]. Arbuscular mycorrhizal fungi -mediated response is a multi-faceted process of drought-responsive gene expression and activation [70]. These metabolic compounds reduce the osmotic ability and hence leaf water capacity in plants subjected to drought. Arbuscular mycorrhizal fungi plants overcome oxidative stress triggered by a deficiency of water by promoting antioxidant compounds to scavenge ROS and facilitate enzyme antioxidant activities [71, 72]. Arbuscular mycorrhizal fungi root colonization enhances root formation, hydraulic properties and root design, resulting in a highly efficient root system for water nutrients absorption [73]. In a previous report, *G*. *mosseae* and *G*. *deserticola* demonstrated improved infectivity when AMF spores were treated by storages in various soil water capacities [74, 75], implying that their consistency in root colonization could have good impacts.

Arbuscular mycorrhizal fungi has shown correct water intakes and improves plant nutrition by hyphal elongation in a drought stress analysis. Besides, enhanced water status will contribute to increased root and hydraulic conductivity. Arbuscular mycorrhizal fungi perseverance to regenerate a linked network after facing a water deficit stress, particularly anastomosis to the disrupted mycelium [76, 77].

Silicon, notably water deficits, has shown positive results on agriculture to mitigate plant-inherent abiotic tensions. This is because of its polymerization in the cell walls of roots, stems and leaves after absorption and accumulation, which creates a double layer of silica [78, 79]. Thus, an enzyme similar to plant protection mechanisms improves the cell walls’ strength and steepness, decreases perspiration, and increases peroxidase activity. Under field conditions, Si facilitated greater dehydration tolerance and turgor loss by preserving the water content in cells and increasing photosynthesis production [80, 81]. The mechanism of abiotic stress tolerance in Si influenced several researchers investigated plants. Silicon’s usage increases plant resistance to water deficits. Spatial benefits to dry plants may be attributed, in part, to its positive impact on the water state and photosynthesis of plants. It has been verified that Si also affects inorganic phosphorus leaf concentration [33, 82]. Proline is an amino acid that avoids the drought and tension in plants due to salinity [83], retains osmotic changes [84], metabolises antioxidants [85], modulates reactive oxygen species [86], and preserves the cell membrane’s stability [87]. Mauad, Crusciol [88], Raza, Haider [89], however, finding that a proline concentration is increased in wheat leaves, triggering symptoms alongside the plant and not as a source of resistance to water stress incorporating silicon decreases proline accumulation. A lower degree of chlorophyll or unchanged water stress for other animals was recorded depending on the water deficit and severity.

Similarly, the reduction in proline concentrations induced by AMF suggests that AMF colonization mitigated water stress. The impaired production of amino acid proteins was detected under leaf water stress and measured by a concomitant accumulation of free AA with a reduced protein concentration. The protein storage allows the plant to sustain the water level of leaves [90], mitigate the harmful effects of active and reactive oxygen species in severe and long-term drought [91], and preserve leaves’ water status. In the leaves and roots, water stress releases antioxidant enzymes. However, this activation was insufficient to protect plants against ROS, which is well manifested in increasing concentrations of MDA in water-stressed. The application of AMF and Si to the plants has also increased the antioxidant defense enzyme’s operation (particularly of SOD) [92, 93]. In stressed plants, however, there is no direct biochemical connection between Si and antioxidant capacity. Biochemical improvement of antioxidant resistance pathways was considered a desirable physical result of cell membrane Si-deposition [32, 94]. Some researchers contend that the implication of Si in plant metabolism is caused by Si-induced improvements in antioxidant enzyme activity and amounts of non-enzymatic antioxidant substances in plants subjected to abiotic stress [42, 95].

## Conclusions

*Brassica juncea* L., often known as Indian mustard, is a well-known member of the Brassicaceae family of plants. The availability of water is a significant constraint on the cultivation of this crop. We examined the effects of water shortage on *B*. *juncea* plants with and without silicon and arbuscular mycorrhizal fungus in 8 different treatments in an open field environment (RCBD). The plant reaction to stress was measured using peroxidase, superoxide dismutase, and catalase activities. The best outcomes were obtained from combining Si and AMF under normal as well as under stress conditions. With this information a new water saving technology for *B*. *juncea* may be developed.

## Supporting information

S1 File (XLSX)
